# Skin Graft

**DOI:** 10.1155/2012/563493

**Published:** 2012-02-06

**Authors:** Ruka Shimizu, Kazuo Kishi

**Affiliations:** Department of Plastic and Reconstructive Surgery, Keio University, School of Medicine, 35 Shinanomachi, Shinjukuku, Tokyo 160-8582, Japan

## Abstract

Skin graft is one of the most indispensable techniques in plastic surgery and dermatology. Skin grafts are used in a variety of clinical situations, such as traumatic wounds, defects after oncologic resection, burn reconstruction, scar contracture release, congenital skin deficiencies, hair restoration, vitiligo, and nipple-areola reconstruction. Skin grafts are generally avoided in the management of more complex wounds. Conditions with deep spaces and exposed bones normally require the use of skin flaps or muscle flaps. In the present review, we describe how to perform skin grafting successfully, and some variation of skin grafting.

## 1. Background

Skin graft is one of the most indispensable techniques in plastic surgery and dermatology. Since Reverdin first performed skin autotransplantation in 1869 [1], many pioneers have tried to improve the results of grafting [2–4]. In 1929, Brown et al. established their technique of split-thickness skin grafting, and they differentiated between full-thickness, intermediate-thickness, and epidermal (Thiersch) grafts, pointing out the advantages and disadvantages of each. These fundamental principles of skin grafting still hold true today [5, 6].

 Skin grafts are used in a variety of clinical situations, such as traumatic wounds, defects after oncologic resection, burn reconstruction, scar contracture release, congenital skin deficiencies, hair restoration, vitiligo, and nipple-areola reconstruction [7–9]. Skin grafts are generally avoided in the management of more complex wounds. Conditions with deep spaces and exposed bones normally require the use of skin flaps or muscle flaps.

In the present review, we describe how to perform skin grafting successfully, and some variation of skin grafting.

## 2. Operative Indication

### 2.1. Split-Thickness or Full-Thickness Grafts

Skin grafts are generally classified as split-thickness or full-thickness grafts. When a graft includes only a portion of the dermis, it is called a split-thickness skin graft. When a graft contains the entire dermis, it is called a full-thickness skin graft. Split-thickness skin grafts are further classified into mesh skin grafts, stamp skin grafts, and chip skin grafts, based on their shape [10, 11].

The amount of dermis included with the graft determines both the likelihood of survival and the level of contracture. That is to say, split-thickness grafts can survive in conditions with less vascularity, but they have a greater likelihood of contracture. In contrast, full-thickness grafts require a better vascular bed for survival but undergo less contracture [12].

### 2.2. Donor Sites

**Figure 1:**
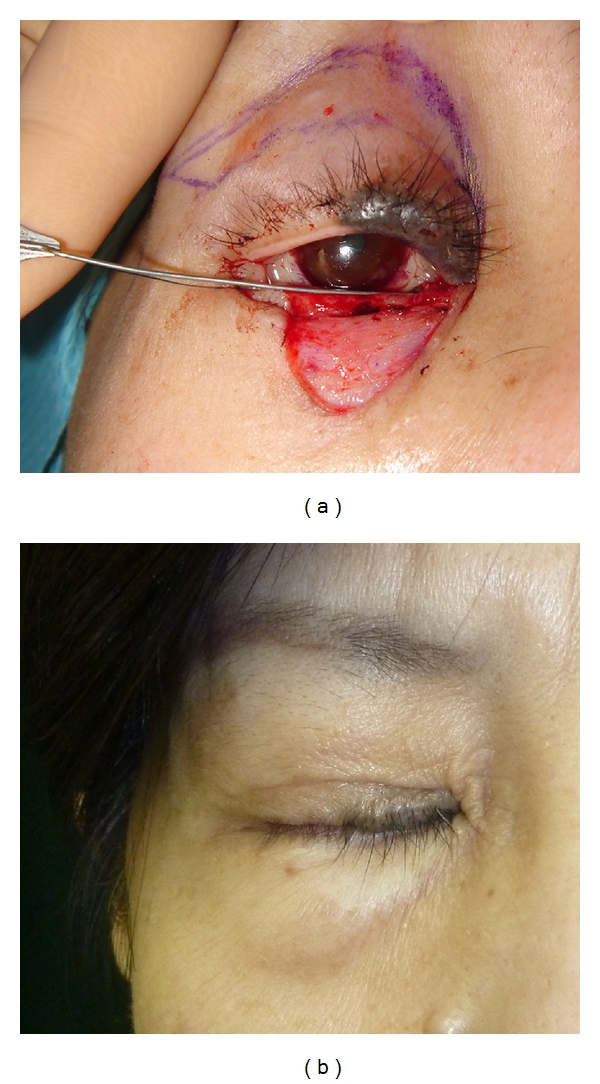
A case of 47-year-old-woman suffering from basal cell carcinoma on her lower eyelid. After resection of the tumor, full-thickness skin grafting was performed from both sides of upper eyelids (a). Two years after operation (b).

With consideration of aesthetic results, the donor site should be similar to the recipient site in terms of consistency, thickness, color, and texture. To cover a facial defect, in which maximum care must be taken, full-thickness skin grafts are often needed. Common donor sites for full-thickness skin grafts of the head and neck include the postauricular region, anterior auricular region, nasolabial crease, supraclavicular region, eyelids, and neck. Even on the same face, skin characteristics may vary widely by location. For example, while eyelid skin is thin and has few glandular structures, nasal skin is thick and has a relatively large number of glandular elements. Though full-thickness skin from the postauricular region is often used to cover defects of the lower eyelid, aesthetic results may be much better if it is possible to use a surplus of upper eyelid skin (Figure 1).

In the other sites of the body, groin region or lower abdomen is frequently used as a donor site because the enough skin can be obtained and the donor site can be primarily sutured. Also, the scar of the donor site can be hidden in the under wear.

**Figure 2:**
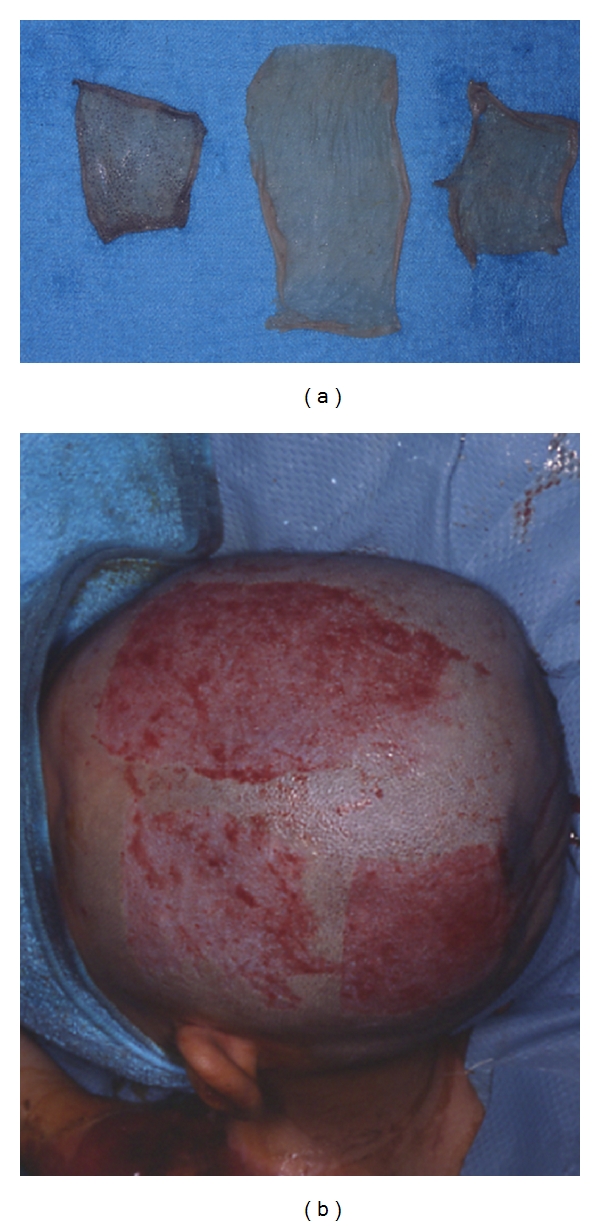
Images of split-thickness skin taken from the head at 350 *μ*m in thickness (a) and the donor site (b).

Split-thickness skin grafts may be taken from any area of the body, including the scalp (Figure 2). Despite its ability to heal spontaneously, the donor site of a split-thickness skin graft is frequently scarred or discolored. If the patient agrees with hair shaving, it is effective to take grafts from a hair-bearing region, because the scarring after skin harvesting can be hidden in the hair. In addition, re-epithelialization is faster, because of the remaining rich hair follicles. When taking a graft from a hair-bearing region, it is important to take a thin graft, because thicker split-thickness grafts will contain undesired hair follicles and eventually lead to hair in the graft and hair loss in the donor site. We usually harvest split-thickness skin from the scalp less than 350 *μ*m in thickness. For men, the donor site should be chosen with care based on the potential for hair regression by male pattern baldness.

### 2.3. The Location of the Recipient Site

Everywhere where effective blood microcirculation is observed, skin grafts have a chance to take. Thus, it is possible to do skin grafting on the granulation tissue, dermis, adipose tissue, fascia, muscle, periosteum, perichondrium, and paratenon. On the contrary, it is difficult to do skin grafting on the surface of the bone, cartilage, and tendon. When the skin graft must be applied on the bone, cortical bone is abraded and cancellous bone is exposed. On the cancellous bone, skin graft can be taken. When the skin defect is small in these areas, artificial dermis can be applied, and skin may be grafted secondarily.

## 3. Operative Procedure

### 3.1. Skin Harvesting

In harvesting full-thickness skin grafts, generally the scalpel is used. All of the dermis is included in the graft with as little subcutaneous fat as possible. Another way is to remove the fat with scissors after harvesting skin including fat. The donor site is then closed primarily.

 Split-thickness skin grafts can be harvested by power-driven dermatome, drum dermatome, and free-hand dermatome. A free-hand dermatome offers a quick method of harvesting a skin graft that does not depend on electricity or pneumatic power; thus, it is useful in harvesting small and thin grafts. However, it is difficult to control the exact thickness and depth of the graft with a free-hand dermatome. Since Brown and McDowell first introduced the electrically driven dermatome in 1949 [6], the motorized dermatome has largely replaced the free-hand dermatome for large split-thickness harvests, because of its simplicity and reliability. Infiltration of the subcutaneous tissue with saline prior to using a motorized dermatome can facilitate skin graft harvest, especially when harvesting skin over a bony prominence [13]. Also, lubrication with a small amount of Vaseline ointment makes it easier to harvest the skin by decreasing the friction between the skin and the dermatome. The donor site of split-thickness skin grafts is covered with wound dressing materials to moisten the environment of the wound.

 There is a unique method called “Suction Blister Therapy,” which is often used in the treatment of vitiligo. With this method, only the epidermis is harvested, and epidermal cells including pigment cells are grafted to the shaved surface of the vitiligo.

**Figure 3:**
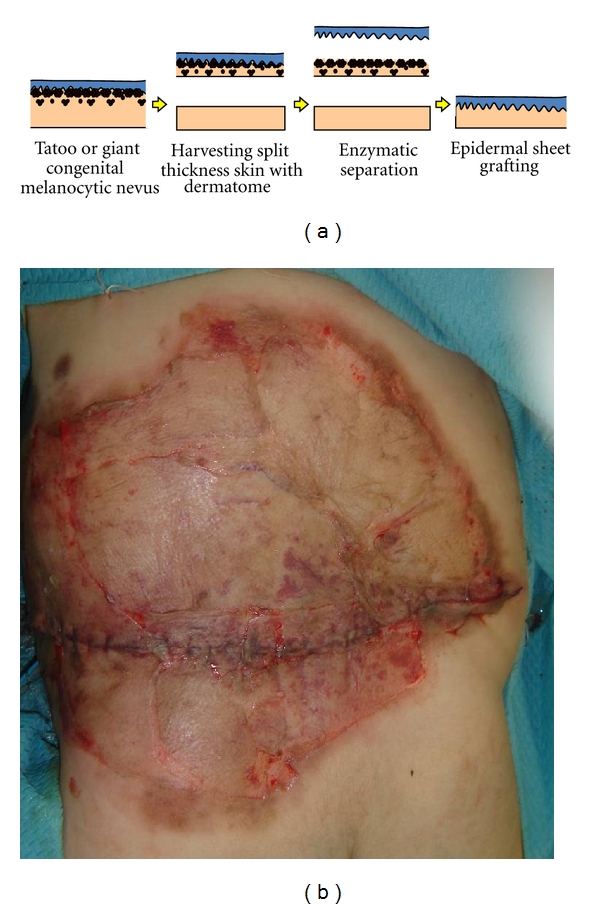
Images of enzymatically separated epidermal sheet grafting (a). Two-month-old girl with giant congenital melanocytic nevi on her back. Enzymatically separated epidermal sheet was grafted after discarding upper dermis (b).

Also, in some situations, such as in the treatment of a tattoo or giant congenital melanocytic nevi, only the upper dermis is discarded, and the enzymatically separated epidermis can be used (Figure 3) [14].

**Figure 4:**
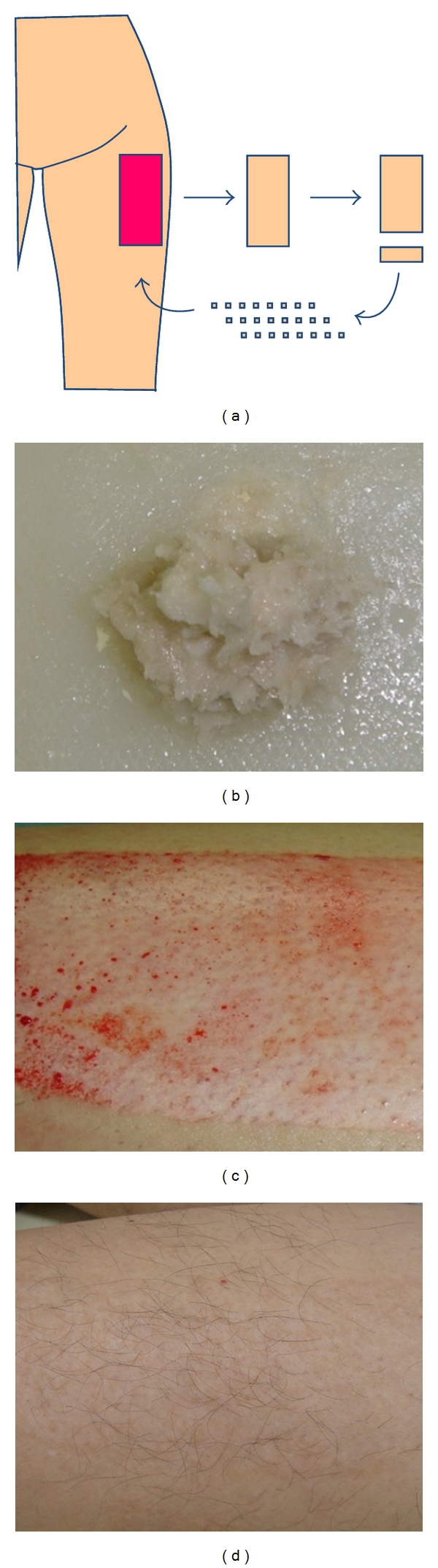
Image of recruited minced skin grafting (a), and minced skin (b). The donor site before minced skin was transplanted (c). One year after minced skin grafting (d). Scar is almost invisible.

The optimal treatment of a donor site is autografting [15]. When excess skin is available after grafting, it can be placed onto the donor site rather than discarded. We add a step to mince the graft to the extent to which the skin splinters are not visible, which is called “recruited minced skin grafting” (Figure 4).

### 3.2. Graft Preparation

When the defect to be grafted is extensive or has convoluted surfaces, split-thickness skin grafts can be meshed to expand. This meshing process not only increases the surface area that can be covered by the harvested graft but also allows the graft to adhere better to a convoluted wound [16]. The disadvantages of meshed grafts are wounds with a checkerboard appearance, which leaves an aesthetically less attractive scar, and the possibility of causing more contraction of the wound. Also, it is important to note that meshing a skin graft does not prevent postoperative bleeding. The only way to prevent graft loss from a hematoma is the achievement of hemostasis.

### 3.3. Graft Fixation

Before the dressing is applied, the graft should be inspected for hematoma formation. Flushing beneath the graft with saline removes blood clots and provides for better adherence of the graft. In graft fixation, the first step is to apply a nonadherent dressing. Then, a dressing should be applied to the graft with gentle pressure (10–20 mmHg) to promote graft adherence without causing pressure necrosis [17]. “Tie-over dressing” is useful, because it minimizes the risk of hematoma or seroma formation and also prevents shearing forces from outside. If a mesh skin graft is going to be applied, or if a secondary operation is planned over the good granulation tissue on the surface of the wound bed, it may be sufficient to place cotton balls or press fluffed gauze onto the wound after suturing the graft. When the tie-over dressing is needed at an infectious site, it is better to remove the fixing suture earlier on 2-3 days after operation and observe the grafted skin. Many fixation techniques have been reported, such as reverse tie-over fixation, use of a wire frame or stoppers in tie-over fixation or quilting sutures, use of fibrin glue and dermabond, and negative pressure dressing without tie-over dressing [18–21].

### 3.4. Postoperative Care for Preventing Complication

The most common complications with skin grafts are skin pigmentation and skin graft contraction. The thinner the graft, the more often these complications tend to occur. However, even if full-thickness skin grafts are used, for example, a graft from groin to palm, irreversible pigmentation may remain which is not acceptable. Hydroquinone cream is useful for treating transient pigmentation. Skin graft contraction is of particular concern when split-thickness skin grafts are used on the flexor side of joints or in the palm. In those cases, immobilization with a splint or other device is very important.
